# Health-economic evaluation of meniscus tear treatments: a systematic review

**DOI:** 10.1007/s00167-022-07278-8

**Published:** 2023-01-13

**Authors:** R. Deviandri, M. C. Daulay, D. Iskandar, A. P. Kautsar, A. M. T. Lubis, M. J. Postma

**Affiliations:** 1 Department of Orthopedics, University of Groningen, University Medical Center Groningen, Hanzeplein 1, 9713 GZ Groningen, The Netherlands; 2 Department of Physiology, Faculty of Medicine, Universitas Riau, Pekanbaru, Indonesia; 3 Division of Orthopedics, Arifin Achmad Hospital, Pekanbaru, Indonesia; 4 Faculty of Pharmacy, Universitas Bhakti Kencana, Bandung, Indonesia; 5 Faculty of Pharmacy, Universitas Padjadjaran, Bandung, Indonesia; 6 Unit of Global Health, Department of Health Sciences, University Medical Center Groningen (UMCG), University of Groningen, Groningen, The Netherlands; 7 Department of Orthopedics, Faculty of Medicine, Universitas Indonesia/Cipto Mangunkusumo Hospital, Jakarta, Indonesia; 8 Department of Economics, Econometrics & Finance, Faculty of Economics & Business, University of Groningen, Groningen, The Netherlands; 9 Department of Pharmacology & Therapy, Universitas Airlangga, Surabaya, Indonesia; 10 Center of Excellence in Higher Education for Pharmaceutical Care Innovation, Universitas Padjadjaran, Bandung, Indonesia

**Keywords:** Meniscus injury, Cost, Cost analysis, Economic analysis, Meniscus tear, Systematic review

## Abstract

**Purpose:**

To evaluate the overall evidence of published health-economic evaluation studies on meniscus tear treatment.

**Methods:**

Our systematic review focuses on health-economic evaluation studies of meniscus tear treatment interventions found in PubMed and Embase databases. A qualitative, descriptive approach was used to analyze the studies’ results and systematically report them following PRISMA guidelines. The health-economic evaluation method for each included study was categorized following one of the four approaches: partial economic evaluation (PEE), cost-effectiveness analysis (CEA), cost–benefit analysis (CBA), or cost-utility analysis (CUA). The quality of each included study was assessed using the Consensus on Health Economic Criteria (CHEC) list. Comparisons of input variables and outcomes were made, if applicable.

**Results:**

Sixteen studies were included; of these, six studies performed PEE, seven studies CUA, two studies CEA, and one study combined CBA, CUA, and CEA. The following economic comparisons were analyzed and showed the respective comparative outcomes: (1) meniscus repair was more cost-effective than arthroscopic partial meniscectomy (meniscectomy) for reparable meniscus tear; (2) non-operative treatment or physical therapy was less costly than meniscectomy for degenerative meniscus tear; (3) physical therapy with delayed meniscectomy was more cost-effective than early meniscectomy for meniscus tear with knee osteoarthritis; (4) meniscectomy without physical therapy was less costly than meniscectomy with physical therapy; (5) meniscectomy was more cost-effective than either meniscus allograft transplantation or meniscus scaffold procedure; (6) the conventional arthroscopic instrument cost was lower than laser-assisted arthroscopy in meniscectomy procedures.

**Conclusion:**

Results from this review suggest that meniscus repair is the most cost-effective intervention for reparable meniscus tears. Physical therapy followed by delayed meniscectomy is the most cost-effective intervention for degenerative meniscus tears. Meniscus scaffold should be avoided, especially when implemented on a large scale.

**Level of evidence:**

Systematic review of level IV studies.

**Supplementary Information:**

The online version contains supplementary material available at 10.1007/s00167-022-07278-8.

## Introduction

Meniscus tears are the most prevalent and treated injuries in the knee joint, with a bimodal age distribution in young-active people and older people. Meniscus tear incidence is estimated at 60 per 100,000, although this number is likely underestimated [1]. Jarraya et al. found that over 75% of patients with symptomatic osteoarthritis have a meniscus tear [2]. Meniscus tear surgery is one of the most routinely performed orthopaedic procedures in the orthopedic field, with high annual costs [3]; therefore, early diagnosis and appropriate treatment for meniscus tears are increasingly crucial in current orthopedic research [1, 4].

Options to treat meniscus tears fall into two general categories: non-operative and operative management; the latter can be divided into three main methods: meniscectomy, either partial or total; meniscus repair; and meniscus transplantation, either meniscus scaffold or meniscus allograft transplantation [3]. Both host factors (e.g., age, co-morbidities, and compliance) and tear characteristics (e.g., location of tear/age/reducibility of tear) need to be considered before selecting the most appropriate treatment [5]. Evidence suggests that degenerative tears in older patients (age > 40) without mechanical symptoms can be effectively treated non-operatively with a structured physical therapy program as a first-line option. On the other hand, meniscus repair is more suitable for younger patients (age < 40) with peripheral reducible tears *(e.g.,* nearer the capsular attachment) of the horizontal or longitudinal pattern and shows 80% success at two years [5, 6]. However, symptomatic tears not amenable to repair should be treated with meniscectomy, as meniscus function can still be preserved, especially when the peripheral meniscus rim is intact [5, 7].

As the concept of “value-based care” increasingly emerges, all aspects of health practice need to be re-evaluated to maintain health services’ affordability and sustainability. According to the Economist Intelligence Unit, value-based healthcare is “the creation and operation of a health system that explicitly prioritizes health outcomes which matter to patients relative to the cost of achieving this outcome” [8]. Hence, aggressive, preventive or curative interventions which are often costly but deliver outcomes with high effectiveness and efficiency are needed. Lowering the cost of treatments by sacrificing results is not an option in value-based healthcare [9]; therefore, understanding the cost drivers and high-value procedures within orthopedics is paramount if value-based health care is to be applied to this specialty. Specifically, the focus is to identify high-volume procedures with clear transparent choices and criteria and determine the value of these interventions.

A systematic review is important to look at the treatment of meniscus tears from a health-economic perspective. This study aimed to evaluate the evidence of published health-economic evaluation studies on meniscus treatment interventions. The health-economic studies associated with the procedure were identified, the data available were summarized, and the cost-effectiveness strategy among the procedures was determined.

## Materials and methods

### Overview and eligibility criteria for review

This systematic review was reported using Preferred Reporting Items for Systematic Reviews and Meta-Analyses (PRISMA) guidelines. It was registered in PROSPERO (Registration number: CRD42021262185). All economic studies published on PubMed and Embase databases were identified up to 30 April 2022. The following inclusion criteria were used: (1) cost analysis was performed on the meniscus tear’s treatment procedure; (2) based on either an economic model or a trial; and (3) clinical relevance to meniscus treatment. Analyses that did not report meniscus treatment-related cost values were excluded from the review. Studies issued as commentary, editorials, research protocols, and reviews, and studies not written in English were excluded.

### Search method for identification of studies and data collection

A systematic search was conducted in two major electronic databases, PubMed and EMBASE. The references of the included studies were then reviewed to expand the search further and identify relevant publications. The search had no limitation for date of publication.

The search strategy was developed using the patient/population, intervention, comparison and outcomes (PICO) approach, which includes thesaurus and accessible terms related to or describing the condition and outcome. The PICO model was selected for its known relevance to defining clinical questions based on patients’ specific problems and the research question.

After reviewing the titles and abstracts, studies were included or excluded based on the inclusion criteria. Next, the full text was retrieved for further review for studies needing further inquiry into their inclusion status. Two authors (RD and MD) reviewed the full text of eligible studies for further data extraction. Review inconsistencies were resolved by joint review and consensus between reviewers.

### Data synthesis and qualitative analysis of studies

All outcome variables reported in the included studies were extracted into the pre-specified data extraction form. Given the heterogeneity of the existing evidence, a qualitative, descriptive approach was used to assess the pooled results from the economic studies on meniscus treatment. The economic evaluation method of each included study was categorized into one of four approaches based on the availability of (1) comparison of two or more alternative interventions and (2) comparison of the costs and effects of the treatments in each study. The types of economic evaluations are partial economic evaluation (PEE), cost-effectiveness analysis (CEA), cost–benefit analysis (CBA), and cost-utility analysis (CUA) [10]. PEE measures disease cost without intervention comparison and does not relate costs to outcome. The costs of each treatment are analyzed straightforwardly in terms of monetary costs, assuming equal health outcomes for each intervention. CEA estimates the outcomes expressed in a natural health unit, such as number of patients with clinical improvement, cures, and life-years gained. The results of such comparisons may be stated either in terms of incremental cost per unit of effect. CBA measures and compares each intervention in terms of benefit and cost, all aspects expressed in monetary units. The results of CBA express the consequences of an intervention in monetary terms in order to facilitate comparison to program costs. CUA–often the preferred technique–measures and compares each intervention in terms of cost and utilities, indicating preferences for health outcomes using a generic measure of health gain, synthesized in cost per quality-adjusted life-year (QALY) [10, 11].

Regarding financial implications, comparisons were made for the following cost-related variables associated with the procedure: meniscus repair, arthroscopic partial meniscectomy (meniscectomy), physical therapy, non-operative treatment, meniscus allograft transplantation, meniscus scaffold, and other unique comparisons reported in a single study. Further, the type of meniscus tears behind these procedures was also classified as either reparable or irreparable meniscus tears. Costs included in this systematic review were converted to 2020 US dollars ($) using CCEMG-EPPI-Centre Cost Converter v.1.6 (accessible online at https://eppi.ioe.ac.uk/costconversion/), as suggested by Mastrigt et al. [11, 12].

A narrative data synthesis was performed by presenting all findings in summative form, including tables and figures. Primary outcomes were the average costs, effectiveness, and incremental cost-effectiveness ratio (ICER). Secondary outcome was the type of economic evaluation and the specificities of each study.

### Quality assessment of studies

The CHEC list, which comprises 19 questions, was used to investigate the methodological quality of the economic studies by two independent reviewers (DI and AK). Each question is assigned either a “yes” or “no”. The two reviewers evaluated the papers and confirmed or disconfirmed compliance with each assessment question. Any disagreement was resolved by a joint review and consensus between reviewers. Each question in the CHEC list was rated with three possible answers: *N* = no, with no points; *U* = unclear, with half a point; and *Y* = yes, with one point. As the CHEC list does not define the summary scores specifically, we defined the score limits for the methodological quality of the studies; a total score ≥ 14.5 was considered a high-quality economic evaluation, a total score of 10–14 a moderate-quality economic evaluation, and a score < 10 a low-quality evaluation [8, 11, 13]. The agreement levels of interobserver assessment were determined by kappa value.

Statistical analyses were performed using SPSS Statistics version 26.0 (IBM).

## Results

### Study selection

**Fig. 1:**
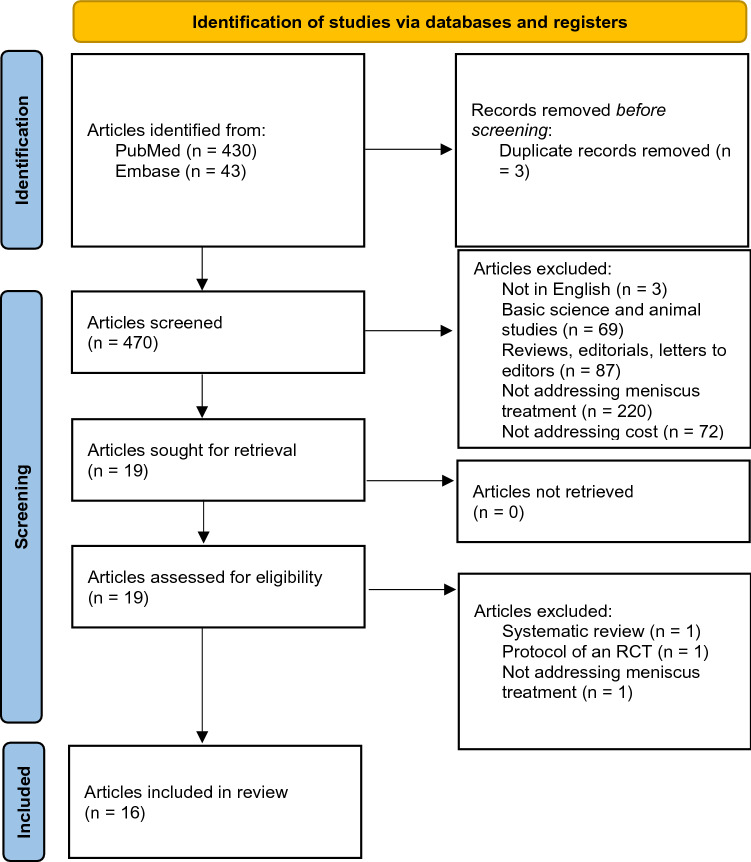
Study flow diagram as PRISMA guidelines[21].

The search strategy identified 473 studies; after review of these initially selected studies, 16 were considered eligible for inclusion in our study (Fig. 1).

### General characteristics of included studies

The general characteristics of the included studies are presented in Table 1. A total of 16 studies were identified and included; six performed PEEs [7, 14–18], seven CUAs [6, 19–24], two CEA [25, 26], and one study performed a CUA, CEA, and CBA simultaneously [12]. Five studies compared meniscus repair vs meniscectomy [6, 7, 19, 20, 22], three studies compared costs of meniscectomy vs non-operative treatment [14, 17, 24], two studies compared physical therapy with delayed meniscectomy vs early meniscectomy [12, 21], two studies directly compared the costs of having vs not having physical therapy after a meniscectomy [15, 16], three studies investigated meniscus transplantation, and one study compared costs associated with conventional arthroscopic instruments vs laser-assisted treatment in meniscectomy [18].

**Table 1:** General characteristics of included studies

Author, year of publication	Country	Studies’ specificities						Type of economic evaluation	Study design	Time horizon	Outcome measure
		Gender	Sample sizes	Age	Cost year	Perspective	Interventions				
Feeley et al. 2016 [6]	USA	M, F	Not specified	20–80	2014	Health care	MR, APM	CUA	Model-based (Markov)	30 years	Cost/QALY
Lester et al. 2018 [20]	USA	Not specified	Not specified	Not specified (young age)	2017	Health care	MR, APM	CUA	Model-based (Markov)	40 years	Cost/QALY
Rogers et al. 2019 [22]	USA	Not specified	Not specified	Not specified (young age)	2018	Health care	MR, APM	CUA	Model-based (Markov)	40 years	Cost/QALY
Faucett et al. 2019 [19]	USA	M, F	Not specified	20–80	2017	Health care	MR, APM,, NO	CUA	Model-based (Markov)	10 years	Cost/QALY
Sochacki et al. 2020 [7]	USA	M, F	27,580 (M: 16,218, F: 11,362)	29.9 ± 15.1	2016	Health care	MR, APM	PEE	Trial-based (observational)	10 years	Cost
Rongen et al. 2016 [23]	Netherlands	M, F	Not specified	Not specified (older age)	2015	Societal	APM, NO	CUA	Model-based (Markov)	9 years	Cost/QALY
Barnds et al. 2019 [14]	USA	M, F	290,601 (M: 120,309, F: 170,292)	Not specified	2016	Health care	APM, NO	PEE	Trial-based (obervational)	10 years	Cost
Hershman et al. 2020 [17]	USA	M, F	50 (M:34, F:16)	55.98 ± 8.46	2018	Health care	APM, NO	PEE	Trial-based (observational)	4 years	Cost
Forster et al. 1982 [15]	UK	M	86	16–45	1977	Societal	PT, non-PT	PEE	Trial-based (RCT)	4 years	Cost
Goodwin et al. 2005 [16]	UK	M, F	84 (M:72, F:12)	18–60	2005	Societal	PT, non-PT	PEE	Trial-based (observational)	1.5 years	Cost
Ramme et al. 2016 [26]	USA	F	Not specified	25–30	2014	Health care	APM, MAT	CEA	Model-based (Markov)	25 years	Cost/ rate to TKA
Rongen et al. 2018 [24]	Netherlands	M, F	Not specified	Not specified	2016	Societal	APM, MS	CUA	Model-based (Markov)	1 year (model 1); 5 years (model 2)	Cost/QALY
Bendich et al. 2018 [25]	USA	Not specified	Not specified	20–50	2017	Health care	MAT, NO	CEA	Model-based (Markov)	30 years	Cost/ rate to OA
Losina et al. 2015 [21]	USA	Not specified	Not specified	58 ± 7	2013	Societal	PT, delayed APM,, immediate APM	CUA	Model-based (Markov)	10 years	Cost/QALY
van de Graaf et al. 2020 [12]	Netherlands	M, F	319 (M:158, F:161)	45–70	2016	Societal	APM, PT	CEA, CUA, CBA	Trial-based (RCT)	2 years	Cost/QALY, cost/IKDC
Yakin et al. 1999 [18]	USA	M, F	84 (M:67, F:26)	15–76	1994	Health care	conventional arthroscopic instrument, laser-assisted	PEE	Trial-based (observational)	4 years 5 months	Cost

*MR* meniscus repair, *APM* arthroscopic partial meniscectomy, *PT* physical therapy, *MAT* meniscus allograft transplantation, *MS* meniscus scaffold, *CUA* scst-utility analysis, *PEE* partial economic analysis, *CEA* cost-effectiveness analysis, *CBA* cost–benefit analysis, *QALY* quality-adjusted life-year, *TKA *total knee arthroplasty, *IKDC* international knee documentation committe, *RCT* randomized controlled trial, *M* male, *F* female

The included studies varied geographically; 11 studies were conducted in the United States [6, 7, 14, 17–22, 25, 26], three studies in the Netherlands [12, 23, 24], and two in the United Kingdom [15, 16].

Methodological quality of the studies was assessed using the CHEC list and is displayed in Table 2. CHEC scores of the included studies ranged from 8 to 19. Agreement levels of interobserver assessment ranged from − 0.09 to 1 of kappa value. The entire agreement of each point of CHEC is reported in Appendix Table A 1–2.

**Table 2:** CHEC list scoring of the included studies

Author, Year of Publication	Mean score	1st reviewer	2nd reviewer	κ value
Barnds et al. 2019 [14]	17.5	18	17	0.16
Bendich et al. 2018 [25]	10.5	14	7	− 0.15
Faucett et al. 2019 [19]	16.5	17	16	0.46
Feeley et al. 2016 [6]	17	18	16	− 0.09
Forster et al. 1982 [15]	17	18	16	0.38
Goodwin et al. 2005 [16]	8	10	6	0.41
Hershman et al. 2020 [17]	11	14	8	0.66
Lester et al. 2018 [20]	12.5	11	14	0.56
Losina et al. 2015 [21]	14.5	14	15	1
Ramme et al. 2016 [26]	19	19	19	− 0.09
Rogers et al. 2019 [22]	17	18	16	0.56
Rongen et al. 2016 [23]	14.5	15	14	0
Rongen et al. 2018 [24]	18	19	17	− 0.07
Sochacki et al. 2020 [7]	17.5	18	17	0.28
van de Graaf et al. 2020 [12]	13	12	14	0
Yakin et al. 1999 [18]	18.5	18	19	0.29

*CHEC* consensus health-economic criteria

### Meniscus repair vs meniscectomy

Five studies compared meniscus repair vs meniscectomy. Two studies performed a direct comparison [6, 7], one study with additional non-operative treatment after medial meniscus root tears [19], one in the setting of ACL reconstruction [20], and another specifically involving red–red zone repair or meniscectomy [22]. In a large database study, Sochacki et al. [7] found that a meniscus repair costs more than a meniscectomy ($7,680 vs $5,871). However, the other four studies showed meniscus repair to be a more cost-effective treatment: Feeley et al. [6] compared meniscus repair vs meniscectomy (ICER $806/QALY vs $975/QALY), Faucett et al. [19] compared the treatment of meniscus root tear cases between meniscus repair, meniscectomy, and non-operation (ICER $3,483/QALY vs $5,127/QALY vs $3,969/QALY), Rogers et al. [22] compared isolated meniscus repair vs meniscectomy for the red–red zone (ICER $1,185/QALY vs $2,362/QALY), and Lester et al. [20] compared meniscus repair vs meniscectomy in the setting of anterior cruciate ligament reconstruction (ICER $1,056/QALY vs $1,533/QALY).

### Meniscectomy vs non-operative treatment

Three studies compared meniscectomy vs non-operative treatment, two using PEE [14, 17] and one using CUA [24]. The two studies agreed that meniscectomy generated more cost than not operating ($3,993 vs $427, and $4,740 vs $1,862, respectively) [14, 17]. In addition, Rongen et al. [24] showed the result of ICER $3,574/QALY vs $2,740/QALY between meniscectomy and non-operative treatment.

### Early meniscectomy vs physical therapy with delayed meniscectomy

Of the studies that compared early meniscectomy vs physical therapy with delayed meniscectomy, one examined patient with a non-obstructive meniscus tear [12]; one investigated patient with a meniscus tear in knee osteoarthritis [21]. Van de Graaf, et al. concluded that early meniscectomy was less cost-effective than physical therapy with delayed meniscectomy (ICER $83,047/QALY), with non-inferiority margins of 0.89 for QALY [12]; and Losina, et al. found that early meniscectomy was less cost-effective than physical therapy with optional delayed meniscectomy (ICER $116,320/QALY) [21].

### Physical therapy vs non-physical therapy in a meniscectomy setting

Two PEE studies investigated physical therapy after meniscectomy [15, 16]. One stated that the mean cost per patient of providing outpatient physical therapy was $120 [15], the other compared the cost incurred by the group that had physical therapy with the group that did not ($3,906 vs $3,576) [16].

### Meniscus transplantation

Two studies discussed meniscus allograft transplantation [25, 26], one demonstrating that it needs to be approximately one-third more effective in delaying osteoarthritis in previously meniscectomized knees to be cost-effective and stating that the mean costs of meniscus allograft transplantation are higher than those of non-operative treatment ($8,714 vs $3,061, respectively) [25]. The other study stated that meniscus allograft transplantation had been shown to reduce pain and improve function in patients with a discoid lateral meniscus tear and postponed total knee arthroplasty (TKA) rate for more years than meniscectomy, yet a meniscus allograft transplantation is more costly than meniscectomy ($16,007 vs $11,538, respectively) [26]. One study on CUA compared the cost-effectiveness of meniscus scaffold vs meniscectomy, showing that meniscus scaffold was less cost-effective than meniscectomy both for a lifetime (ICER $73,445/QALY) and for a five-year period (ICER $401,492/QALY) [23].

### Unique comparison data

One PPE study discussing conventional instruments vs laser-assisted meniscectomy concluded that conventional meniscectomy was recommended for routine intervention as the cost was lower ($1,796 vs $2,503, respectively) [18]. The results of the included studies are presented in Table 3.

**Table 3:** Results of included studies with adjusted costs to 2020 value

Author, Year of Publication	Procedure	Meniscus type	Meniscus reparability	Outcome				Discount rate
				Average cost	Average effectiveness (QALY)	ICER	Conclusion	
Feeley et al. 2016 [6]	MR vs APM	Meniscus tear, reparable	Reparable	MR: $13,325 APM: $15,962	MR: 16.53 APM: 16.37	$72,444	MR dominant (per-patient cost savings $2637)	3%
Lester et al. 2018 [20]	MR vs APM	Peripheral longitudinal tear at either the red-white or red-red zone with an ACL tear	Reparable	MR: $19,016 APM: $26,315	MR: 18.00 APM: 17.16	$8,689.45	MR dominant (per-patient cost savings $8689.45)	NA
Rogers et al. 2019 [22]	MR vs APM	Red-red zone, vertical tears in young adults	Reparable	MR: $24,883 APM: $40,157	MR: 21 APM: 17	$15,273.8	MR dominant (per-patient cost savings $4088.61)	NA
Faucett et al. 2019 [19]	MR vs APM vs NO	Medial meniscus root tears with no osteoarthritis	Reparable	MR: $24,001 APM: $33,497 NO: $26,568	MR: 6.89 APM: 6.53 NO: 6.69	NA	MR more dominant than APM and NO (per-patient cost savings $1747 and $516, respectively)	3%
Sochacki et al. 2020 [7]	MR vs APM	Any type of meniscus tear	NA	MR: $7680 APM: $5871	NA	NA	MR more costly $1809	NA
Rongen et al. 2016 [23]	APM vs NO	Degenerative meniscus tears and knee osteoarthritis	Irreparable	APM: $23,362 NO: $17,823	APM: 8.09 NO: 8.05	$204,220	APM less cost-effective	NA
Barnds et al. 2019 [14]	APM vs NO	Any type of meniscus tear	NA	APM: $3993 NO: $427	NA	NA	APM more costly $3566	NA
Hershman et al. 2020 [17]	APM vs NO	Medial meniscus deficiency (degenerative and/or torn medial meniscus and/or previous meniscectomy)	Irreparable	APM: $4663 NO: $4252	NA	NA	APM more costly $411	NA
Forster et al. 1982 [6]	PT vs non-PT	Medial meniscus tears after APM	Irreparable	PT: $120	NA	NA	PT more costly	NA
Goodwin et al. 2005 [16]	PT vs non-PT	Any type of meniscus tear after APM	Irreparable	PT: $3906 Non-PT: $3576	NA	NA	PT more costly $330	NA
Ramme et al. 2016 [26]	MAT vs APM	Discoid lateral meniscus tears	Irreparable	MAT: $16,007 APM: $11,538	MAT: 17.30 years to TKA APM: 12.50 years to TKA	$931	MAT more effective and more costly	3%
Rongen et al. 2018 [24]	MS vs APM	Irreparable medial meniscus injury	Irreparable	Lifetime term: MS: $39,985 APM: $31,906 5-year term: MS: $20,542 APM: $7293	Lifetime term:MS: 23.69 APM: 23.575-year-term: MS: 3.81 APM: 3.78	Lifetime term: $73,445 5-year-term: $401,492	MS less cost-effective for both lifetime and 5-year term	4%
Bendich et al. 2018 [25]	MAT vs NO	Any type of meniscus tear after total or subtotal meniscectomy	Irreparable	MAT: $8714 NO: $3061	NA	NA	MAT more costly $5653. MAT needs to be 31% more effective in delaying OA compared to NO in order to be more cost-effective	3%
Losina et al. 2015 [21]	PT vs delayed APM vs immediate APM	Degenerative meniscus tear in the presence of knee osteoarthritis	Irreparable	PT: $12,173 Delayed APM: $13,413 Immediate APM: $14,540	PT: 6.63 Delayed APM: 6.72 Immediate APM: 6.73	Delayed APM vs PT: $14,540 Immediate APM vs delayed APM: $116,320	Delayed APM is the most cost-effective	NA
Van de Graaf et al. 2020 [12]	PT with delayed APM vs Immediate APM	Non-obstructive meniscus tears	Irreparable	PT with delayed APM: $5306 Immediate APM: $8079	PT with delayed APM: 1.65 Immediate APM: 1.68	PT with delayed APM: $83,047 per QALY lost	PT with delayed APM less cost-effective. PT non-inferior to APM 0.89 for QALY	4%
Yakin et al. 1999 [18]	Conventional instrument vs laser-assisted APM	Any type of meniscus tear	NA	Conventional instrument: $1796 Laser-assisted: $2503	NA	NA	Conventional instrument less costly by $707	NA

*MR* meniscus repair, *APM* arthroscopic partial meniscectomy, *PT* physical therapy, *MAT* meniscus allograft transplantation, *MS* meniscus scaffold, *ACL* anterior cruciate ligament, *QALY* quality-adjusted life-year, *ICER* incremental cost-effectiveness ratio, *TKA* total knee arthroplasty

## Discussion

This study evaluates the evidence of published health-economic evaluation studies on meniscus tear treatment. The most important finding of the present study was cost-effectiveness of meniscus tear treatment depending on type of meniscus tear. Meniscus repair is more cost-effective for reparable meniscus tears, while physical therapy with delayed meniscectomy is more cost-effective for irreparable and degenerative meniscus tears.

This review showed that meniscus repair instead of meniscectomy; non-operative treatment instead of meniscectomy; physical therapy with delayed meniscectomy instead of early meniscectomy; meniscectomy without physical therapy vs meniscectomy with physical therapy; meniscectomy instead of meniscus allograft transplantation; and conventional instrument instead of laser-assisted meniscectomy gave more value. It was also concluded that meniscus scaffold should be avoided, especially when implemented on a large scale.

Surgery is usually recommended for most meniscus tears except those causing minor symptoms in less active patients, and is urgently recommended for locked knees [1, 5, 27, 28]. Treatment aims to preserve as much functional meniscal tissue as possible. Clinical symptoms caused by meniscus tears should also be addressed. However, room for discussion is still wide open about whether surgery’s increased benefits outweigh the higher costs. Non-operative treatment may be offered for a meniscus tear in less mobile, passive, and less demanding patients, as symptoms may be minimal or uncommon [1, 5, 29].

It is possible to classify meniscus tears into two broad categories: reparable and irreparable tears. Reparable tears present in young patients with horizontal tears in the vascular zone plus longitudinal and radial tears, and are usually traumatic cases [6, 30]. Tears are irreparable if they occur in the avascular zone or complex pattern, and most are degenerative cases [2, 29]. Repairs are generally performed in mild arthrosis (KL grade ≤ 2), mild varus alignment, and a chondral injury grade ≤ grade 2. Poor prognosis is predicted in repairs done on severe cartilage degeneration and severe varus malalignment [31]. The studies included in this systematic review followed the standard procedure of treatments for meniscus tears and lesions, choosing repairs for reparable tears and meniscectomy and/or other non-operative modalities for irreparable tears. This needed to be emphasized, as it proved that the included studies did not discard the importance of accurate and evidence-based treatment choice, although the focus was on cost-effectiveness.

Sochacki et al. concluded that meniscus repair costs more than meniscectomy [7]. This is most likely due to initial differences in implant cost [19, 32]. Day-of-surgery costs are also higher in meniscus repair than in meniscectomy [19]. Meniscus repair becomes more cost-effective after 10 and 30 years of operation because the rate of knee osteoarthritis and TKA is lower [6]. Physical therapy followed by delayed meniscectomy is the treatment of choice for degenerative meniscus tear. Losina et al. [21] and van de Graaf et al. [12] investigated degenerative types of meniscus tear for presence of knee osteoarthritis and horizontal-type tear with complex degenerative meniscus, respectively. Both papers showed that physical therapy followed by delayed meniscectomy is more cost-effective than early meniscectomy for degenerative meniscus tear.

The results found in this study regarding traumatic and degenerative meniscus lesions are in line with those of a previous study describing meniscus repair as the preferred strategy for traumatic meniscus injury, and physical therapy followed by delayed meniscectomy as the preferred intervention for degenerative meniscus tear [29, 30]. The findings from the health-economic perspective could support the development of recommendations for clinical practice guidelines in this field, explicitly considering health-economic evidence such as costs and cost-effectiveness.

The previous health-economic review describing meniscus scaffold and meniscal allograft as likely more effective than meniscectomy for medial meniscus injury and lateral discoid meniscus tear is being in line with the findings [26, 33]. This is because meniscus scaffold interventions are more effective in reducing pain and improving function, and postpone the rate of TKA for longer than meniscectomy [23, 26]. However, using the standard threshold of $50,000 as a basis of the cost-effectiveness strategy, meniscus scaffold is a less cost-effective strategy than meniscectomy, with an ICER of $73,445/QALY for a lifetime and an ICER of $401,492/QALY for five-year time horizon [23].

This review included either model-based or trial-based studies, both of which are mutually supportive and provide prominent evidence for health-economic assessments. A trial-based study presents direct and exact evidence in a particular field. However, because the time horizon of such a study is limited, its results should be used with reservations. A longer time horizon is favored for a health-economic assessment, which could be resynthesized by a model-based study. Although guided by trial-based studies in small populations, clinicians, payers, and regulators are likely to use a model-based study to sharpen their decision-making.

The risk of bias needs to be assessed in the included studies, as bias can overestimate or underestimate the actual intervention effect. There are available tools to evaluate the risk of bias in economic evaluations. Mastrigt et al. explain that the CHEC checklist is a preferred option for appraising trial-based and model-based economic evaluations [10]. Most of the included studies had an appropriate score on the CHEC list, so the findings of this systematic review can be considered low in risk of bias while providing valuable information to support health technology assessment in this field.

This review has some limitations. First, the heterogeneity and diversity of all included data could result in bias. Although the majority of included studies were considered of moderate-to-high-quality economic evaluation, the CHEC score of all included data is broad, ranging from 8 to 19. The level of agreement from − 0.09 to 1.0 of the kappa values showed some disagreement between the reviewers. The kappa value is frequently used to access interrater reliability and represents the extent to which the data collected in the study are correct representations of the variable measured [34]. However, the judgments about the acceptable kappa value for health research should be contingent on the researcher’s perception of what is relevant to the field of research. In this review we still included studies with a low kappa value, thanks to the critical evidence of the included studies [34].

Next, indirect cost assessment in health care is typically measured through loss of productivity and absenteeism. However, only six out of 16 included studies assessed this cost element, so we could not provide indirect cost reporting. Accordingly, recommendations on a more cost-effective treatment based on meniscus type could not be accurately formulated as there were various types of menisci and pre-existing knee conditions. Plus, only a limited number of studies were investigated. Some topics were discussed in a single economic analysis, including using a meniscus scaffold to treat meniscus tears. Hence, strong recommendations on this topic cannot be provided. Last, the results of studies conducted in the USA and Europe could not be transferable to other countries due to the diversity of healthcare systems. Studies in other regions are, therefore, needed.

This study showed the most cost-effective treatment for some types of meniscus tears. Clinicians can use the findings of this review in their day-to-day practice by considering each patient’s type of meniscus tear in their decision-making process. Although costs alone should not drive decision-making, but cost-effectiveness should be taken into consideration during discussion of treatment options with the patient.

## Conclusion

Various meniscus treatment modalities are available for the treating surgeon to choose, broadly classified into meniscus repair, meniscectomy, non-operative approach with or without physical therapy, and meniscus transplantation. Results from this review conclude that meniscus repair is the most cost-effective intervention for reparable meniscus tears, while the physical therapy with delayed meniscectomy is the preferred strategy for degenerative meniscus tears. At the same time, meniscus scaffold should be avoided, especially when implemented on a large scale.

## Supplementary Information

Below is the link to the electronic supplementary material.

Supplementary file1 (DOCX 30 KB)

## Data Availability

The authors declare that the data supporting the findings of this study are available within the article [and its supplementary information files].
