# Supplementary material for: Health-economic evaluation of meniscus tear treatments: a systematic review
Source: Knee Surg Sports Traumatol Arthrosc. 2023 Jan 13;31(9):3582–93. doi: 10.1007/s00167-022-07278-8 (PMC10435400; doi:10.1007/s00167-022-07278-8)
Supplement: Supplementary file 1 — Supplementary file1 (DOCX 30 KB) [file 167_2022_7278_MOESM1_ESM.docx]

Appendix Table A.1 CHEC List scoring of the included studies (Author DI)

| Author, Year of Publication | Q1 | Q2 | Q3 | Q4 | Q5 | Q6 | Q7 | Q8 | Q9 | Q10 | Q11 | Q12 | Q13 | Q14 | Q15 | Q16 | Q17 | Q18 | Q19 | Sum |
| --- | --- | --- | --- | --- | --- | --- | --- | --- | --- | --- | --- | --- | --- | --- | --- | --- | --- | --- | --- | --- |
| Barnds et al., 2019 [2] | 1 | 1 | 1 | 1 | 1 | 1 | 1 | 1 | 1 | 1 | 1 | 1 | 1 | 1 | 1 | 1 | 0 | 1 | 0 | 17 |
| Bendich et al., 2018 [3] | 1 | 1 | 1 | 1 | 0 | 0 | 0 | 0 | 0 | 0 | 0 | 0 | 0 | 0 | 0 | 0 | 1 | 1 | 1 | 7 |
| Faucett et al., 2019 [9] | 1 | 1 | 1 | 1 | 1 | 1 | 1 | 0 | 1 | 1 | 0 | 0 | 1 | 1 | 1 | 1 | 1 | 1 | 1 | 16 |
| Feeley et al., 2016 [10] | 1 | 1 | 1 | 1 | 1 | 1 | 1 | 0 | 1 | 1 | 1 | 1 | 1 | 1 | 1 | 1 | 1 | 0 | 0 | 16 |
| Forster et al., 1982 [11] | 1 | 1 | 1 | 1 | 1 | 1 | 1 | 0 | 1 | 1 | 1 | 0 | 1 | 1 | 1 | 1 | 1 | 0 | 1 | 16 |
| Goodwin et al., 2005 [12] | 1 | 1 | 1 | 0 | 0 | 0 | 0 | 0 | 0 | 0 | 0 | 1 | 0 | 0 | 0 | 1 | 1 | 0 | 0 | 6 |
| Hershman et al., 2020 [13] | 1 | 1 | 1 | 1 | 0 | 1 | 1 | 1 | 0 | 0 | 0 | 0 | 0 | 0 | 0 | 1 | 0 | 0 | 0 | 8 |
| Lester et al., 2018 [18] | 1 | 1 | 1 | 1 | 1 | 0 | 1 | 1 | 1 | 1 | 1 | 1 | 0 | 0 | 0 | 1 | 1 | 1 | 0 | 14 |
| Losina et al., 2015 [19] | 1 | 1 | 1 | 1 | 1 | 1 | 1 | 0 | 1 | 1 | 1 | 1 | 1 | 0 | 1 | 1 | 0 | 1 | 0 | 15 |
| Ramme et al., 2016 [27] | 1 | 1 | 1 | 1 | 1 | 1 | 1 | 1 | 1 | 1 | 1 | 1 | 1 | 1 | 1 | 1 | 1 | 1 | 1 | 19 |
| Rogers et al., 2019 [28] | 1 | 1 | 1 | 1 | 1 | 1 | 1 | 0 | 1 | 1 | 0 | 0 | 1 | 1 | 1 | 1 | 1 | 1 | 1 | 16 |
| Rongen et al., 2016 [29] | 1 | 1 | 1 | 1 | 1 | 0 | 1 | 0 | 1 | 1 | 1 | 1 | 0 | 0 | 1 | 1 | 1 | 1 | 0 | 14 |
| Rongen et al., 2018 [30] | 1 | 1 | 1 | 1 | 1 | 1 | 1 | 0 | 1 | 1 | 1 | 1 | 1 | 1 | 1 | 1 | 0 | 1 | 1 | 17 |
| Sochacki et al., 2020 [31] | 1 | 1 | 0 | 1 | 1 | 1 | 1 | 0 | 1 | 1 | 1 | 1 | 1 | 1 | 1 | 1 | 1 | 1 | 1 | 17 |
| van de Graaf et al., 2020 [32] | 1 | 1 | 1 | 1 | 1 | 1 | 1 | 0 | 0 | 1 | 1 | 1 | 0 | 0 | 0 | 1 | 1 | 1 | 1 | 14 |
| Yakin et al., 1999 [34] | 1 | 1 | 1 | 1 | 1 | 1 | 1 | 1 | 1 | 1 | 1 | 1 | 1 | 1 | 1 | 1 | 1 | 1 | 1 | 19 |

Abbreviation: Q, question

Appendix Table A.2 CHEC List scoring of the included studies (Author AK)

| Author, Year of Publication | Q1 | Q2 | Q3 | Q4 | Q5 | Q6 | Q7 | Q8 | Q9 | Q10 | Q11 | Q12 | Q13 | Q14 | Q15 | Q16 | Q17 | Q18 | Q19 | Sum |
| --- | --- | --- | --- | --- | --- | --- | --- | --- | --- | --- | --- | --- | --- | --- | --- | --- | --- | --- | --- | --- |
| Barnds, Brandon et.al., 2019 | 1 | 1 | 1 | 1 | 1 | 1 | 1 | 1 | 1 | 1 | 1 | 1 | 1 | 1 | 1 | 1 | 1 | 1 | 0 | 18 |
| Bendich, Ilya et.al., 2018 | 1 | 1 | 1 | 0 | 0 | 1 | 1 | 1 | 1 | 1 | 1 | 1 | 0 | 0 | 0 | 1 | 1 | 1 | 1 | 14 |
| Faucett, Scott C. et.al., 2019 | 1 | 1 | 1 | 1 | 1 | 1 | 1 | 1 | 1 | 1 | 1 | 1 | 1 | 0 | 1 | 1 | 0 | 1 | 1 | 17 |
| Feeley, Brian T. et.al., 2016 | 1 | 1 | 1 | 1 | 1 | 1 | 1 | 1 | 1 | 1 | 1 | 1 | 1 | 1 | 1 | 1 | 1 | 1 | 0 | 18 |
| Forster, D. P. et.al., 1982 | 1 | 1 | 1 | 1 | 1 | 1 | 1 | 1 | 1 | 1 | 1 | 1 | 1 | 1 | 1 | 1 | 1 | 1 | 0 | 18 |
| Goodwin, Peter Charles et.al., 2005 | 1 | 1 | 1 | 0 | 0 | 0 | 1 | 1 | 1 | 1 | 1 | 1 | 0 | 0 | 0 | 1 | 0 | 0 | 0 | 10 |
| Hershman, Elliott B. et.al., 2020 | 1 | 1 | 1 | 1 | 0 | 1 | 1 | 1 | 1 | 1 | 1 | 1 | 0 | 0 | 0 | 1 | 1 | 0 | 1 | 14 |
| Lester, Jonathan D. et.al., 2018 | 1 | 1 | 1 | 0 | 0 | 0 | 1 | 1 | 1 | 1 | 1 | 1 | 0 | 0 | 0 | 0 | 1 | 1 | 0 | 11 |
| Losina, Elena et.al., 2015 | 0 | 1 | 1 | 1 | 1 | 0 | 1 | 1 | 1 | 1 | 1 | 1 | 1 | 0 | 1 | 1 | 0 | 1 | 0 | 14 |
| Ramme, Austin J. et.al., 2016 | 1 | 1 | 1 | 1 | 1 | 1 | 1 | 1 | 1 | 1 | 1 | 1 | 1 | 1 | 1 | 1 | 1 | 1 | 1 | 19 |
| Rogers, Mark et.al., 2019 | 1 | 1 | 1 | 1 | 1 | 1 | 1 | 1 | 1 | 1 | 1 | 1 | 1 | 1 | 1 | 1 | 1 | 1 | 0 | 18 |
| Rongen, J. J. et.al., 2018 | 0 | 1 | 1 | 1 | 1 | 0 | 1 | 1 | 1 | 1 | 1 | 1 | 1 | 0 | 1 | 1 | 1 | 1 | 0 | 15 |
| Rongen, Jan J. et.al., 2016 | 1 | 1 | 1 | 1 | 1 | 1 | 1 | 1 | 1 | 1 | 1 | 1 | 1 | 1 | 1 | 1 | 1 | 1 | 1 | 19 |
| Sochacki, Kyle R. et.al., 2020 | 1 | 1 | 1 | 1 | 1 | 1 | 1 | 1 | 1 | 1 | 1 | 1 | 1 | 1 | 1 | 1 | 1 | 1 | 0 | 18 |
| van de Graaf, Victor A. et.al., 2020 | 1 | 1 | 1 | 0 | 0 | 0 | 1 | 1 | 1 | 1 | 1 | 1 | 0 | 0 | 0 | 1 | 1 | 1 | 0 | 12 |
| Yakin, David E. et.al., 1999 | 1 | 1 | 1 | 1 | 0 | 1 | 1 | 1 | 1 | 1 | 1 | 1 | 1 | 1 | 1 | 1 | 1 | 1 | 1 | 18 |

Abbreviation: Q, question

Appendix Table A.3 The agreement levels of interobserver assessment

| Author, Year of Publication | Agreement |
| --- | --- |
| Barnds, Brandon et.al., 2019 | 52.63% |
| Bendich, Ilya et.al., 2018 | 73.68% |
| Faucett, Scott C. et.al., 2019 | 89.47% |
| Feeley, Brian T. et.al., 2016 | 78.95% |
| Forster, D. P. et.al., 1982 | 68.42% |
| Goodwin, Peter Charles et.al., 2005 | 68.42% |
| Hershman, Elliott B. et.al., 2020 | 84.21% |
| Lester, Jonathan D. et.al., 2018 | 84.21% |
| Losina, Elena et.al., 2015 | 100.00% |
| Ramme, Austin J. et.al., 2016 | 78.95% |
| Rogers, Mark et.al., 2019 | 84.21% |
| Rongen, J. J. et.al., 2018 | 89.47% |
| Rongen, Jan J. et.al., 2016 | 84.21% |
| Sochacki, Kyle R. et.al., 2020 | 68.42% |
| van de Graaf, Victor A. et.al., 2020 | 94.74% |
| Yakin, David E. et.al., 1999 | 63.16% |
